# Effectiveness and safety of polyacrylamide hydrogel injection for knee osteoarthritis: results from a 12-month follow up of an open-label study

**DOI:** 10.1186/s13018-024-04756-2

**Published:** 2024-05-02

**Authors:** Henning Bliddal, Jannie Beier, Andreas Hartkopp, Philip G. Conaghan, Marius Henriksen

**Affiliations:** 1 The Parker Institute, Bispebjerg Frederiksberg Hospital, University of Copenhagen, Ndr. Fasanvej 57, 2000 Copenhagen, Frederiksberg, Denmark; 2 Rheumatolog i Odense, Odense, Denmark; 3 A2 Rheumatology and Sports Medicine, Holte, Denmark; 4 Leeds Institute of Rheumatic and Musculoskeletal Medicine, University of Leeds & NIHR Leeds Biomedical Research Centre, Leeds, UK

**Keywords:** Osteoarthritis, Polyacrylamide, WOMAC, Pain, Knee, Intra-articular

## Abstract

**Objective:**

There are few effective osteoarthritis (OA) therapies. A novel injectable polyacrylamide hydrogel (iPAAG) previously demonstrated efficacy and safety up to week 26 in an open-label study of knee OA. Here we report longer-term effectiveness and safety data.

**Methods:**

This multi-centre, open-label study included patients with symptomatic and radiographic knee OA. Primary outcome was WOMAC pain (0–100 scale) at 13 weeks, and patients continued to 26 weeks before entering a further 26-week extension phase. Secondary efficacy outcomes included WOMAC stiffness and function subscales, Patient Global Assessment (PGA) and proportion of OMERACT-OARSI responders. Safety outcomes were adverse events (AEs).

**Results:**

49 participants (31 women, mean age 70) received an ultrasound-guided, intra-articular injection of 6 ml iPAAG; 46 completed the extension phase to 52 weeks. There was a significant reduction in the WOMAC pain score from baseline to 52 weeks (− 17.7 points (95% CI − 23.1; − 12.4); *p* < 0.0001). Similar sustained improvements were observed for WOMAC stiffness (11.0 points; 95% CI − 17.0; − 4.9), physical function (18.0 points; 95% CI − 19.1; − 10.6), and PGA (16.3 points; 95% CI − 23.1; − 9.4). At 52 weeks 62.2% of patients were OMERACT-OARSI responders. From 26 to 52 weeks, 8 adverse effects (AE), including 1 serious AE (cerebrovascular accident) were reported in 5 subjects. None of the new adverse events were thought to be device related.

**Conclusion:**

This open-label study suggests persistent benefits and safety of iPAAG through 52 weeks after a single injection.

*Trial registration:* Clinicaltrials.gov NCT04179552.

## Introduction

Osteoarthritis (OA) is the most common form of arthritis, affecting 3.3–3.6% of the population globally [1]. OA is characterized by pathology involving the entire joint, including cartilage degradation, bone remodelling, osteophyte formation, and synovial inflammation, leading to pain, stiffness, swelling, and loss of normal joint function [2]. Risk factors for developing OA include age, female gender, obesity, anatomical factors, muscle weakness, and joint injury (occupation/sports activities) [1].

Treatment goals for OA are to minimize pain and functional loss. Comprehensive management of the disease involves both non-pharmacologic and pharmacologic therapies. Typically, patients with mild symptoms can be managed by non-pharmacologic means such as (1) avoidance of activities exacerbating pain or overloading the joint, (2) exercise to improve strength, (3) weight loss, and (4) occupational therapy for unloading joints via brace, splint, cane, or crutch. Moderate or severe OA symptoms need combination approaches.

Intraarticular (IA) joint injections (such as glucocorticoid injections or hyaluronic acid injections) can be an effective treatment for OA, especially in a setting of acute pain. However, these injections remain a controversial option due to the need for repeated injections and inconclusive data regarding efficacy versus placebo [1, 3–5].

Arthrosamid®, an injectable polyacrylamide hydrogel (iPAAG, Contura Ltd), is a proprietary cross-linked polyacrylamide hydrogel, containing polyacrylamide (2.5%) and non-pyrogenic water (97.5%). The unique molecular structure allows normal water exchange and integration with the surrounding soft tissue without losing its volume. iPAAG is structurally stable and has been used for various indications such as bulking for stress urinary incontinence and soft tissue augmentation for more than 20 years. iPAAG has also demonstrated promising potential as a treatment for symptomatic OA [6, 7].

Upon injection into the joint cavity, iPAAG integrates into the synovial tissue of the inner capsule [8, 9]. The non-absorbable, non-biodegradable and non-migratory characteristics of Arthrosamid® provide durable augmentation of the inner joint capsular tissue [7]. The exact mechanisms of how this provides pain relief in OA patients is being examined.

An observational clinical study in patients with knee OA reported that iPAAG delivered as two injections of 3 ml separated by 1 month provided symptomatic relief up to 13 months [6]. Following these results, the investigators hypothesised whether the observed efficacy could be retained, and the risk of intraarticular infection could be reduced by using a single 6 ml injection.

An open prospective study reported the six-month efficacy and safety results of one injection of 6 ml iPAAG on knee pain in patients with moderate to severe knee OA [7]. This article presents the 1-year data from the extension phase of this open prospective study.

## Methods

This multi-centre, prospective, open-label clinical study of iPAAG in patients with knee OA consisted of a 26-week main study period followed by a further observation at 12 months. The study enrolled patients with a clinical diagnosis of OA and radiographic evidence of mild to severe OA. Full inclusion and exclusion criteria have been reported previously [7].

The study has been performed in accordance with ISO 14155:2020 (Good Clinical Practice).

### Treatment administered

iPAAG was provided in sealed sterile, pre-filled 1 ml syringes and injected into the intra-articular cavity using a sterile 21G × 2-inch (0.8 × 50 mm) needle. Each patient received 6 ml of iPAAG under ultrasound guidance to ensure proper placement inside the joint cavity. Prior to the injection, prophylactic antibiotics were given, and topical anaesthetics were applied. Analgesic treatment with nonsteroidal anti-inflammatory drugs (NSAIDs) and paracetamol was allowed during the trial, but not exceeding the recommended dosage and not within 48 h prior to a study visit. Non-pharmacological therapy was allowed during the study. All the concomitant therapies and treatments were recorded.

### Study endpoints

The endpoints of this extension study were change from baseline to week 52 in the WOMAC pain, stiffness and physical function subscales and the PGA of impact of osteoarthritis. The proportion of OMERACT-OARSI responders was also calculated. The safety endpoints were the incidence of adverse events and adverse device effects.

### Statistical methods

Details of the sample size calculation have been published previously [7]. Briefly, a sample size of 38 was required to obtain a statistical power of 90%. Assuming a dropout rate of 20%, 48 subjects in total were required. Baseline data were defined as the last assessment with available data before the injection.

Changes from baseline to 52 weeks in the WOMAC pain, stiffness and physical function subscales and the PGA were analysed for the ITT analysis set using a mixed model for repeated measurement (MMRM) with a restricted maximum likelihood-based approach. The estimated change based on the least square mean at week 4, week 13, week 26 and week 52 were presented including 95% confidence limits and corresponding p-values. Statistical significance was claimed if the computed p-value was equal to or less than 0.05. No adjustment for multiplicity was done.

AEs and adverse device related effects (ADEs) were presented using descriptive statistics. Only AEs with the start date no later than the week 52 visit were included in this analysis.

## Results

50 participants were screened for this study and 49 were enrolled. Demographic and baseline characteristics are shown for these 49 participants in Table 1. 46 participants completed the 52 weeks assessment (2 withdrawals by participant, 1 due to AE).

**Table 1:** Demographic and baseline characteristics

	Arthrosamid N = 49
*Age (years)*	
Mean (SD)	70.0 (8.6)
Median	72.0
Range	44–86
*Sex (N,%)*	
Female	31 (63.3)
Male	18 (36.7)
*BMI (kg/m*^*2*^*)*	
Mean (SD)	27.5 (3.3)
Median	27.2
Range	21.0–34.6
*Baseline WOMAC pain score (0–100)*	
Mean (SD)	50.3 (11.8)
Median	50.0
Range	20–75
*Baseline WOMAC stiffness score (0–100)*	
Mean (SD)	55.6 (17.5)
Median	62.5
Range	0–88
*Baseline WOMAC phys. function score (0–100)*	
Mean (SD)	46.6 (16.1)
Median	45.6
Range	9–87
*Baseline Patient Global Assessment (0–100)*	
Mean (SD)	61.1 (18.3)
Median	65.0
Range	22–100

*N* number of participants, *SD* standard deviation

**Fig. 1:**
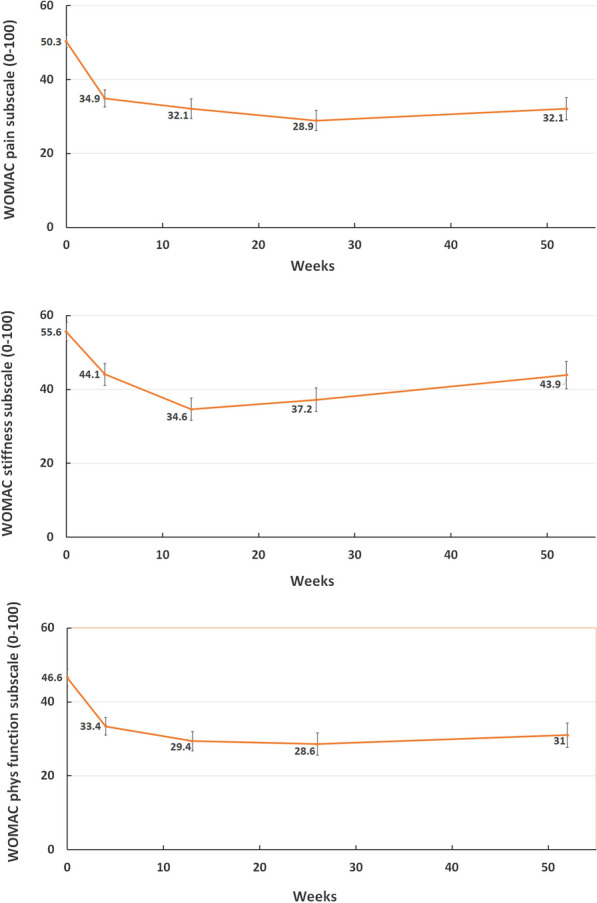
Mean plot transformed WOMAC pain, stiffness and physical function subscales (0–100). Error bars show standard error

Table 2 demonstrates the results from the ITT analysis set for the WOMAC pain, stiffness and physical function subscales and PGA. Figure 1 demonstrates the reduction from baseline to 52-weeks for the WOMAC subscales.

**Table 2:** Change from baseline to week 52 in effectiveness endpoints—ITT population (n = 49)

Endpoint	Mean (95%CI)	*P* value
WOMAC pain	− 17.7 (− 23.1; − 12.4)	< 0.0001
WOMAC stiffness	− 11.0 (− 17.0; − 4.9)	0.0007
WOMAC physical function	− 14.9 (− 19.1; − 10.6)	< 0.0001
Patient Global Assessment	− 16.3 (− 23.1; − 9.4)	< 0.0001

More than half (62.2%) of the participants were positive responders to the OMERACT-OARSI criteria at week 52.

### Safety

Five subjects reported 8 AEs between the 6- and 12-month visits with no event reported more than once. None of the new adverse events were thought to be device related. Seven events were mild while one severe event (cerebrovascular accident) was reported as a serious AE unrelated to treatment. No deaths were reported.

## Discussion

The therapeutic goals of treatment for knee OA include relief of pain, reduction of stiffness, improvement or preservation of joint range of motion, and improvement in or maintenance of mobility, function and health-related quality of life [2]. Ultimately, to delay or avoid the need for total knee replacement surgery would be a desirable outcome. Whilst patients are encouraged to modify lifestyle through diet and exercise, it is clear that for many patients, the effects of lifestyle modification are insufficient to manage pain and allow them to return to a more “normal” level of function. Whilst some injectable interventions are available to treat OA, a novel approach is clearly needed to help manage the growing demographic of older, more obese patients. The backlog of planned operations due to the COVID pandemic has further exacerbated the need for simple, safe and sustaining treatments that can be administered in a clinic setting to reduce the overwhelming load on hospital systems.

The participants of this study can be seen as “typical” knee OA patients and represent the majority of patients presently within the health system seeking help.

The data gathered at 52 weeks show continued effectiveness of iPAAG in reducing the pain and stiffness of the treated knee joint and functional improvement of the knee as compared to baseline levels. Similarly, the mean reduction in the PGA at 52 weeks after treatment with iPAAG continued to reflect an improved quality of life compared to baseline in the treated subjects. It is known that iPAAG acts as a scaffold to integrate and thicken the synovial tissues [8, 9] and it may be that this thickening of the synovial tissue distances the inflammatory cells which may break the inflammatory cycle (which are driving synovial pain within knee OA) thereby reducing the pain experienced by the subject.

The device was well tolerated with few events related to iPAAG reported in the first 6 months [7]. From 6 months to 1 year the device continued to be well tolerated, with only 2 AEs reported as unlikely related to treatment.

Studies examining intra-articular therapy are associated with placebo effects [10]. While this study is limited by the lack of a control group, which introduces a risk of bias to the results, it was designed to detect therapy effects as experienced by the patients and the results are indicative of a considerable duration of benefit following treatment with iPAAG, reflecting similar results seen after the first 26 weeks [7]. Further evidence from a randomized controlled study will be required to confirm these preliminary findings.

## Conclusion

In conclusion, this study in patients with knee OA suggests that a single treatment with 6 ml iPAAG remains effective one year after injection with good clinical effects and no significant safety events. At 52 weeks, the reductions from baseline in the WOMAC pain sub-scale remain clinically significant and suggest that iPAAG may be a promising approach to manage knee OA pain**.** The outcomes will continue to be monitored as part of the ongoing extension phase of this study.

## Data Availability

The dataset used and analysed during the current study are available from the corresponding author on reasonable request.

## Abbreviations

ADEs: Adverse device related effects
AE: Adverse events
IA: Intraarticular
iPAAG: Polyacrylamide hydrogel
NSAIDs: Nonsteroidal anti-inflammatory drugs
OA: Osteoarthritis
OMERACT-OARSI: Outcome measures in rheumatology-OARSI
PGA: Patient global assessment
WOMAC: Western Ontario and McMaster Universities Osteoarthritis Index
